# Detection of long terminal repeat loci derived from endogenous retrovirus in junglefowl using whole-genome sequencing

**DOI:** 10.1038/s41598-023-34520-1

**Published:** 2023-05-06

**Authors:** Shinya Ishihara

**Affiliations:** Department of Animal Science, Nippon Veterinary and Life Science University, 1-7-1 Kyonancho, Musashino, Tokyo 180-8602 Japan

**Keywords:** Evolution, Genetics

## Abstract

Endogenous retroviruses (ERVs) are genetic elements present in the genome that retain traces of past viral infections. Characterization of ERVs can provide crucial insights into avian evolution. This study aimed to identify novel long terminal repeat (LTR) loci derived from ERVs (ERV-LTRs) absent in the reference genome using whole-genome sequencing data of red junglefowl, gray junglefowl, Ceylon junglefowl, and green junglefowl. In total, 835 ERV-LTR loci were identified across the four *Gallus* species. The numbers of ERV-LTRs loci detected in red junglefowl and its subspecies gray junglefowl, Ceylon junglefowl, and green junglefowl were 362, 216, 193, and 128, respectively. The phylogenetic tree was congruent with previously reported trees, suggesting the potential for inferring relationships among past junglefowl populations from the identified ERV-LTR loci. Of the detected loci, 306 ERV-LTRs were identified near or within the genes, and some were associated with cell adhesion. The detected ERV-LTR sequences were classified as endogenous avian retrovirus family, avian leukosis virus subgroup E, Ovex-1, and murine leukemia virus-related ERVs. In addition, the sequence of the EAV family was divided into four patterns by combining the U3, R, and U5 regions. These findings contribute to a more comprehensive understanding of the characteristics of junglefowl ERVs.

## Introduction

Upon retroviral infection, the viral genome is reverse transcribed and integrated into the host genome as a provirus. In principle, the provirus has all the requirements for its replication and consists of an internal region encoding viral genes (*gag*, *pro*/*pol*, and *env*), which are flanked by two identical regulatory long terminal repeats (LTRs) at integration. Adjacent to the provirus is a short target site duplication (TSD) of 4–8 bp in the host genome sequence generated during integration. Vertical transmission can cause such viruses to infect germ cells and reproductive tissues, resulting in the formation of endogenous retroviruses (ERVs) within offspring. Gradually, ERVs can reach a high frequency within populations and eventually become fixed within species^1^. Typical avian ERVs include the avian leukosis virus (ALV) and endogenous avian retrovirus (EAV) families. The ALV family comprises several subgroups, and the ERVs of subgroup E, referred to as ALV-E, often retain high structural integrity^2^. A sequence known as EAV-HP within the EAV family lacks the pol gene, whereas EAV-0 and EAV-51 have the pol gene but lack the env gene^3^. It has been suggested that ALV-E is detected only in *Gallus gallus*, including red junglefowl (*G. gallus gallus*) and commercial chickens, whereas the EAV family could be present across different *Gallus* species^3^.

Approximately 5% of the human genome is derived from ERVs, whereas ERVs constitute approximately 3% of the chicken genome^4,5^. However, there is likely a significant number of ERVs that have not been discovered in chickens. These ERVs have played a role in shaping bird species diversity and have caused economic losses to the poultry industry due to genetic diseases^6–8^. The characterization of ERVs will provide essential insights into avian evolution.

Mitochondrial DNA analysis indicates that the red jungle fowl is an ancestral species of chickens^9,10^. In addition to red junglefowl, three other species belonging to the genus *Gallus* were identified, gray junglefowl (*G. sonneratii*), Ceylon junglefowl (*G. lafayetii*), and green junglefowl (*G. varius*). Red junglefowl is distributed across much of Southeast Asia and parts of South Asia, whereas the other three species have more restricted ranges as follows: gray junglefowl in central and southern India, Ceylon junglefowl in Sri Lanka, and green junglefowl in Java and surrounding islands. Recent molecular genetic studies suggest that various species of *Gallus* contribute to the genetic composition of chicken. However, the origin and history of genetic diversity in chickens remains only partially understood^11–13^. In this study, the aim was to identify the loci of LTR derived from ERV (ERV-LTR) in the genome using whole-genome data for the genus *Gallus*, including subspecies. Additionally, by comparing the ERV-LTR loci among species and the detected sequences of ERV-LTRs, the characteristics of ERV-LTRs from the genus *Gallus* were clarified.

## Results

### Sequencing data quality and identification of the non-reference ERV-LTR breakpoint

Here, 100 bp paired-end reads were mapped using BWA-MEM^14^, and the overall mean sequence depth was 30.6 × (13.5–42.9) for all junglefowls (Table S1). The mapping results are presented in Table S1. More than 95.3% of the paired-end reads for each junglefowl were mapped to the reference *Gallus* genome, whereas only 1.56–31.59% were not properly mapped (improper reads). In addition, 0.10–1.86% of the reads were singletons that were mapped to only one side. The analytical process (see “Methods” section for more detail) was conducted in accordance with the methodology of previous studies^15,16^. The total number of candidate ERV-LTR insertion loci identified for each individual ranged from 39 to 2011 (Table S1) based on RetroSeq software^17^. Next, the Integrated Genome Viewer (IGV)^18^ was employed to confirm the presence or absence of TSDs for all detected loci for each individual. Furthermore, contigs were constructed using the extracted reads from the TSDs and analyzed using blastn^19^. In total, 835 ERV-LTR loci were identified. Most of the identified ERV-LTRs were related to the LTR region of the EAV family (EAV-HP, EAV-51, EAV-0, ev/J, or chicken endogenous LTR). Twenty LTR sequences of ALV-E were identified, and these sequences were present only in red junglefowl and its subspecies (Table S2). In chr2:133,314,053, all species and subspecies had a contig similar to the LTR of the murine leukemia virus (MLV)-related endogenous retrovirus (DQ280312). Moreover, on chr3:54,480,182, all species and subspecies, excluding the green junglefowl, had an Ovex1 (FJ406461). Of the ERVs detected, 306 were present near or within the gene (Table S2). The Gene Ontology (GO) analysis using these gene sets showed six GO terms (Table S3). The top-ranked GO category was “cell adhesion” and included genes such as *RELN*, *CNTN5*, *CDH20*, *CDH7*, *TENM1*, *SPON1*, *NRXN3*, and *CDH4*.

### Comparison of detected ERV loci among species and subspecies

**Figure 1:**
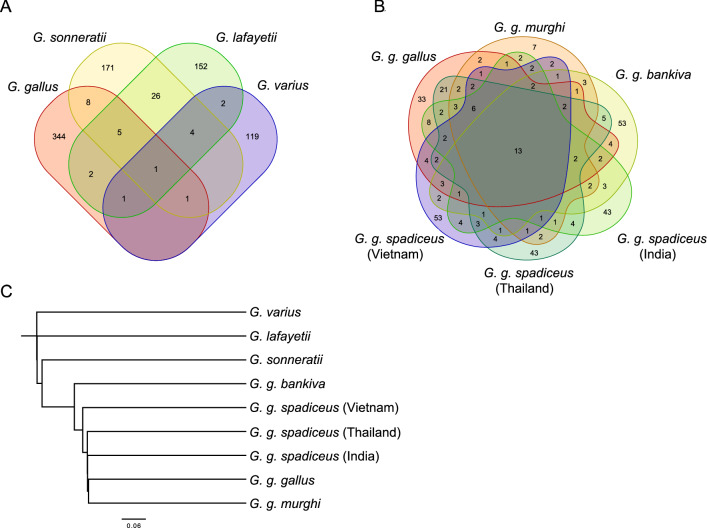
Number of detected endogenous retrovirus (ERV) loci among species and subspecies and phylogenetic tree. (**A**) Venn diagram indicating the number of ERV loci across four species and the overlap between each ERV loci. (**B**) Venn diagram indicating the number of ERV loci across red junglefowl and its five subspecies and the overlap between each ERV loci. (**C**) Phylogenetic tree constructed based on the presence or absence of ERV loci. The bar indicates each distance.

The numbers of ERV loci detected in merged red junglefowl, gray junglefowl, Ceylon junglefowl, and green junglefowl were 362, 216, 193, and 128, respectively (Table 1). The number of ERV loci detected in red junglefowl and its subspecies ranged from to 61–123. The Venn diagram shows ERVs with shared loci among subspecies or species (Fig.1A and B). Among the species, 50 loci were detected as common between two or more species, with gray junglefowl and Ceylon junglefowl exhibiting the highest degree of commonality among species, with 36 loci in common. In contrast, no common loci were detected between green junglefowl and red junglefowl. Among the subspecies of red junglefowl, 13 common ERV loci were detected, with the most common loci identified between the red junglefowl and *Gallus gallus spadiceus* in Thailand, with 57 shared ERVs. The clustering tree created based on all loci is shown in Fig. 1C. The tree branched in the following order: green junglefowl, Ceylon junglefowl, gray junglefowl, and red junglefowl.

**Table 1:** Number of endogenous retrovirus (ERV) loci for each species or subspecies.

Species/subspecies	No. of ERV loci
*G. g. gallus*	116
*G. g. murgha*	61
*G. g. bankiva*	101
*G. g. spadiceus.* I	108
*G. g. spadiceus.* T	123
*G. g. spadiceus.* V	113
*G. sonneratii*	216
*G. lafayetii*	193
*G. varius*	128

*G. g. spadiceus*. I, *G .g. spadiceus*. T, and *G. g. spadiceus*. V were *Gallus gallus spadiceus* from India, Thailand, and Vietnam, respectively. *G. g.*, *Gallus gallus.*

The merged number of detected ERVs in *Gallus gallus* was 362 loci after removing overlap.

### Classification and structure of each intact ERV-LTR

**Figure 2:**
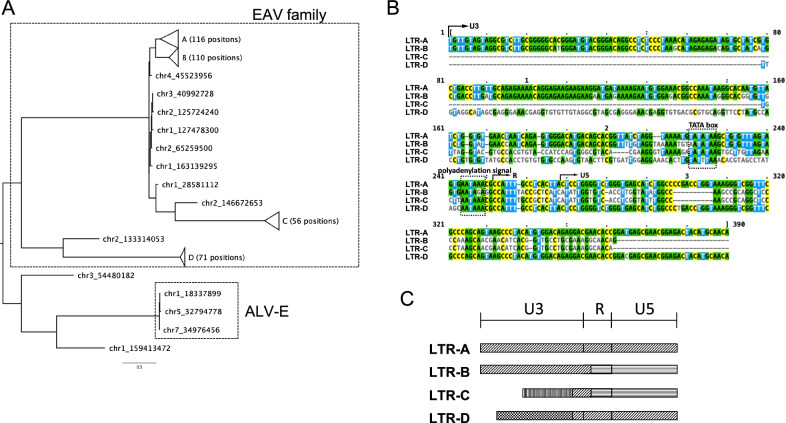
Phylogenetic tree and structure of each intact endogenous retrovirus long terminal repeat (ERV-LTR). (**A**) Phylogenetic tree constructed based on the long terminal repeat sequence. (**B**) Alignment of the representative sequence from four patterns of the endogenous avian virus (EAV) family. (**C**) Schematic diagram of detected EAV-LTR sequence. Identical patterns indicate homologous sequences.

In total, 367 loci that were absent in the reference and possessed TSD sequences on both the 5′ and 3′ flanking contig sequences were obtained. Of these, 79 loci were identified across multiple species or subspecies. These loci and sequences are listed in Table S4. The sequences obtained at the same position were highly similar. For example, among the groups, nine nucleotide substitutions were identified at 346 bp on chr3:99,634,554. Phylogenetic analysis revealed that 362 of these sequences belong to the LTRs of the EAV family. Simultaneously, the remaining five loci were LTRs of the ALV-E, Ovex1, and MLV-related endogenous retroviruses at three, one, and one loci, respectively (Fig. 2A). The LTRs of the EAV family were further divided into four clusters based on their sequence patterns, with the LTR sequences divided into the U3, R, and U5 regions (Fig. 2B and C). LTR-D was consistent with EAV-21-3 (Accession No. AJ6232390). LTR-A shared the R and U5 regions with LTR-D and U3 (consistent with U3 of Accession AJ6232391) with LTR-B. LTR-C was consistent with the sequence of M31065 in all regions. Similarly, LTR-B and LTR-C shared identical R and U5 regions, but the U3 regions were distinct.

## Discussion

After trimming the sequence data obtained in this study based on strict criteria, more than 95% of all read pairs were mapped to the *Gallus* reference genome, although some variation in depth was observed. Thus, the assembled sequence data were considered high quality. In addition, the detection of junglefowl ERV-LTRs was attempted using improper pairs and singleton sequence reads that did not map correctly to the reference genome. In total, 835 ERV-LTR loci were detected in the *Gallus* genome. This result is highly reliable because the presence of TSD was visually confirmed using IGV for the breakpoints detected by RetroSeq, and the contig created by collecting the surrounding sequences also contained ERV-LTR sequences. Previous studies have reported the use of *G. gallus* genomes and next-generation sequencing data for detecting ERV^20,21^. For example, one study utilized obsERVer software in conjunction with the Galgal5 reference genome to detect ALV-E in commercial chickens, resulting in the identification of ALV-E at 20 loci^20^. Similarly, 75.22 ± 9.52 integration sites for EAV-HP were identified in commercial chickens, native chickens, and red junglefowl utilizing Galgal4^21^. Although variations in methodologies and reference genomes make direct comparisons difficult, accumulating such findings will undoubtedly contribute to a more comprehensive understanding of the characteristics of endogenous chicken retroviruses. The RetroSeq-based method used in this study primarily targets non-reference ERV-LTR loci, which, in theory, are excluded from the reference *G. gallus* genome. As a result, 835 non-reference ERV-LTRs were identified at unique genomic positions.

The number of ERV-LTR loci identified in the red junglefowl and its subspecies was relatively low compared with the number of ERV-LTRs detected in other species. This discrepancy could be attributed to the use of the reference genome of the red junglefowl, which might have resulted in an underestimation of the number of ERV-LTRs present, as it does not consider the ERV-LTRs unique to red junglefowl that are already present in the reference genome. Furthermore, the method utilized in this study might not yet be able to detect all non-reference ERV-LTR loci, as a sufficient quantity of improper pairs and singletons is essential for detection. In a specific green junglefowl, a significantly high number of improper pairs (31.59%) was observed. This individual exhibited a higher value (2,011 loci) than other individuals, even after RetroSeq filtering. However, the final ERV-LTR loci identified were not significantly different from those of the others, indicating that a certain threshold of data was adequate for detecting non-reference ERV-LTRs. Nevertheless, of 835 locations obtained, only 367 contigs with TSDs on both sides were obtained. This difference is partly due to the insufficient number of reads, which could be improved to some extent by increasing the data size. Nevertheless, it has been noted that in humans, ERVs have a tendency to accumulate in regions of the genome that are low in complexity and repetitive^22–24^. Moreover, the detection of ERVs containing gag, pol, and env regions, as well as solo-LTRs, poses a challenge when utilizing short-read sequencing. Therefore, the use of long-read sequencing technologies, such as single-molecule real-time sequencing and nanopore sequencing, should be considered to determine the complete insertion sequence.

The ALV family is younger than the EAV family because it is only found in domestic chickens and red junglefowl, whereas the EAV family is restricted to all *Gallus* species^25^. This study detected the LTR derived from the EAV family in all species, whereas that of ALV-E was detected only in red junglefowl and its subspecies, which is consistent with previous reports. Therefore, ALV-E is thought to be an internalized sequence in the red junglefowl genome after divergence of the red junglefowl population from the genus *Gallus*. Species with ERVs at the same locus are thought to have diverged after their common ancestor was infected with a retrovirus, which was internalized. A previous study^26^ estimated the approximate divergence age of the genus *Gallus*. They calculated that red junglefowl and gray junglefowl diverged 2.56 mya red junglefowl and Ceylon junglefowl diverged 2.88 mya, gray junglefowl and Ceylon junglefowl diverged 1.77 mya, and green junglefowl and other *Gallus* species diverged approximately 4.0–4.1 mya. Overall, the phylogenetic tree constructed from the ERV-LTR loci obtained in this study was generally consistent with previously reported phylogenetic relationships. However, it did not reflect the branching age (Fig. 1C).

Three loci (chr3:40,992,728, chr3:101,202,255, and chr11:7,946,729) were not consistent with previously reported phylogenetic relationships. For example, on chr3:101,202,255, ERV-LTRs were detected only in red junglefowl, Ceylon junglefowl, and green junglefowl, but not in gray junglefowl. Such ERV-LTRs might have been lost from the locus through recombination or other mechanisms during speciation. Alternatively, there could have been instances of introgression between the evolutionarily distant species. Previous research has suggested that introgression from green junglefowl to domestic chickens might have occurred on chromosome 5^12^. Additionally, whole-genome data analysis has demonstrated an admixture between green junglefowl and red junglefowl species in Indonesia^26^.

A comparison of the LTR sequences at the same locus revealed nucleotide substitutions among species and subspecies. Additionally, several sequence patterns were observed in the U3, R, and U5 regions of the LTR of the EAV family in this study. This variation could be a consequence of intra-familial recombination, as previously reported^27^. Although these substitutions and LTR variations do not necessarily reflect genetic divergence, they could support the approximation of the complex history of past introgression. Further examination of the dissemination of ERVs across contiguous regions might enhance our understanding of speciation. In contrast to previous phylogenetic analyses based on sequences mapped to a reference genome, this study employed sequences that do not exist in the reference genome, which could facilitate more detailed phylogenetic analyses in conjunction with previous methods.

In the present study, 306 ERV sequences were detected in the genes, some of which were associated with cell adhesion. The presence of ERVs in the chicken genome affects the host. For example, one of the known effects of ERVs on chickens is the blue eggshell phenotype; the *SLCO1B3* gene is expressed in the uterus of hens that lay blue-shelled eggs but not in hens without blue eggshells^8^. An insertion of EAV-HP was identified in the 5′ flanking region of *SLCO1B3*, and in situ hybridization revealed EAV-HP in the 5′ flanking region of *SLCO1B3*^8^. In situ hybridization showed that the EAV-HP insertion was associated with the blue eggshell phenotype. In the present study, LTR insertion into cell adhesion-related genes, such as *RELN*, *CNTN5*, *CDH20*, *CDH7*, *TENM1*, *SPON1*, *NRXN3*, and *CDH4*, was detected. The U3 region of an LTR contains enhancer and promoter sequences that drive viral transcription^28^. It contains other transcription regulatory signals, such as the TATA box^29^. The LTR sequence inserted into *CNTN5* and *NRXN3* contained the TATA box, suggesting that the insertion of ERV-LTRs might have played a role in the evolution of cell adhesion processes. Further research is required to completely understand the mechanisms by which ERV-LTRs influence the evolution of cell adhesion and other biological processes. In addition, if the ERV-LTRs of commercial chickens are nearly as diverse, in terms of ERV polymorphisms, as the ERV-LTRs of junglefowl detected in this study, future ERV analyses of commercial and native chickens could be an important source of genetic novelty for chicken breeding programs.

## Materials and methods

### Whole genome sequence (WGS) data

The WGS data obtained by Illumina HiSeq 2000 or 2500, from a total of 39 individuals^12,30^, including 16 red junglefowls, 8 Gy junglefowls, 10 Ceylon junglefowls, and 5 green junglefowls, were obtained in fastq format from the European Nucleotide Archive (Study Accessions were PRJNA432200 and PRJNA552030). The red junglefowl included the subspecies, three *Gallus gallus murghi*, two *Gallus gallus bankiva*, and seven total *G. g. spadiceus* individuals from populations in India, Thailand, and Vietnam. Accession IDs are listed in Table S1. Nucleotides with low-quality scores in these reads were trimmed, and adapters were removed with Trimmomatic v.0.36 using the ILLUMINACLIP: TruSeq3-PE:2:30:10, LEADING:3, SLIDINGWINDOW:4:20, and MINLEN:30 settings^31^. The reads were mapped to the *G. gallus* reference genome (GRCg6a, GenBank assembly accession: GCF_000002315.6) using Burrows-Wheeler Aligner and Mem algorithms. Data were produced in BAM format.

### Detection of non-reference ERVs

**Figure 3:**
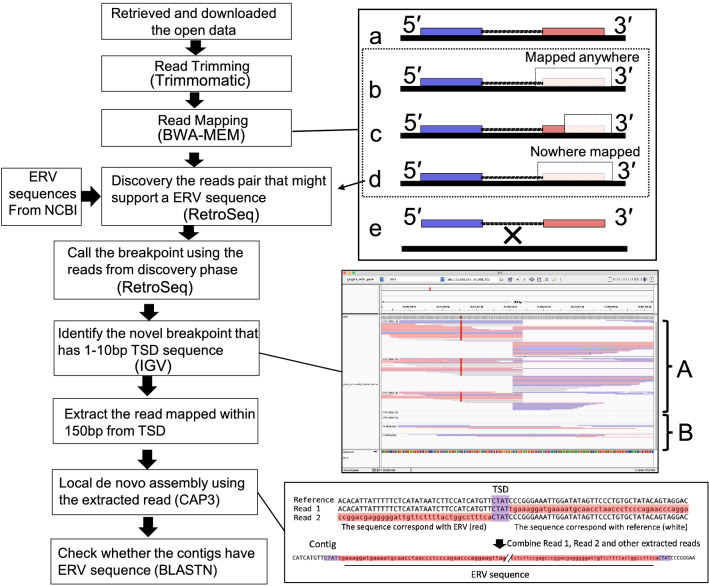
Pipeline for the detection of non-reference *Gallus* endogenous retroviruses-long terminal repeats (ERV-LTRs) in whole-genome sequencing (WGS) read data. In the right-upper panel, the black line denotes the chicken reference genome sequence. Blue and red boxes connected with lines denote the 5′ and 3′ ends of a paired-end sequencing read. Most paired-end reads were identified as proper mapping, whereas a small percentage of them were improper mapping. Proper pair: both ends of the paired-end sequence mapped accurately (**a**). Discordant read and split read: one end of the paired-end sequence mapped accurately, whereas the other end was only partially identified at the expected locus on the reference genome. The unidentified sequence could be mapped anywhere else on the reference genome (**b**, **c**). Singleton: one end of the paired-end sequence mapped accurately, whereas the other end did not map on the reference genome (**d**). Unmapped read pairs: neither read mapped to the reference genome (**e**). Discordant reads, split reads, and singletons were used for RetroSeq analysis. In the right-middle panel, a representative view of the Integrative Genomics Viewer (IGV) is used to confirm the presence of target site duplications (TSDs) at each locus, detected using RetroSeq, extract support reads from the TSD loci, perform local assembly, and analyze the contigs for the presence of endogenous retroviruses (ERV)–genome junctions from both sides. “A” denotes the individuals that have the TSD, and “B” denotes the individuals that did not have the TSD. The right-lower panel shows a conceptual diagram of local de novo assembly using CAP3. Sequences in red indicate sequences not present on the reference genome, and sequences in purple indicate TSDs.

ERV detection was performed according to a previous method^15,16^. The types of read pairs mapped to the reference genome were defined and extracted sequence reads that were useful for this study. Most of the paired-end reads were obtained from the WGS map of the reference genome. However, mismatched read pairs can also occur with unexpected span sizes and orientations. Non-proper pairs are those in which the 5′ or 3′ end maps to a contig sequence in the reference genome and the other end maps entirely or partially to an unexpected locus. A singleton refers to mapping to the reference genome. A singleton refers to one end of a read pair that does not map to the reference genome, whereas an unmapped read pair refers to both ends of a read pair that do not map to the reference genome (Fig. 3). Mismatched read pairs can provide insight into LTR-related loci as anchors. RetroSeq software was used to detect non-reference transposon elements (TEs) using mismatched reads^17^. The process flow is illustrated in Fig. 3. The ERV sequences used for RetroSeq were obtained from the National Center for Biotechnology Information (NCBI, Bethesda, MD, USA) and are listed in Table S5. The reference genome was GRCg6a, which contained only autosomes and sex chromosomes. In the RetroSeq “call” step, TE insertion positions (breakpoints) were estimated using reads detected in the “discover” phase, as previously reported. The call step was set to ≥ 10 to reduce false positives, and the maximum read depth option per call was set to 10,000 to increase the BAM coverage. All other RetroSeq options were used with their default values. A minimum of seven filter-level breakpoints were used. A breakpoint detected within 500 bp was considered identical and excluded. The IGV was used to detect loci containing TSDs. Using the batch script functionality of IGV, a screenshot was obtained at each genomic locus detected by the RetroSeq analysis pipeline and was carefully examined. The loci were presumed to be TSDs if they mapped to reads detected during the “discover” phase either from the 5′ or 3′ side, overlapping by 1–10 bp (Fig. 3). The 5′ and 3′ reads mapped within 150 bp of TSDs were extracted using SAMtools^32^. The extracted read set was used to generate the contig using CAP3 software^33^. The contig sequences obtained using CAP3 were used for a blastn search^19^. The lowest e-value was used to determine the ERV class. Each 200 bp sequence upstream and downstream of the breakpoint was extracted from the reference genome and subjected to blastn to eliminate the possibility of detecting ERV sequences in the reference genome. Loci that matched the ERVs were excluded from the analysis. A sequence not existing in the reference genome was deduced from the contiguous sequence obtained within the region that is delimited on either side by the TSD sequence or the six-base pair sequence on the adjacent 5′ and 3′ sides of the TSD, respectively, if the TSD length was insufficient.

### Analysis of the obtained ERVs

The identified ERV loci were examined for insertions within the gene using IGV. GO analyses for each gene with an ERV sequence were performed using the R package clusterProfiler^34^. The presence or absence of ERVs at each locus was assumed as one or zero for clustering among species and subspecies. The phylogenetic tree based on clustering was generated using the function “dist.binary” with ade4^35^ and “hclust” using the ape^36^ package of R software^37^. The ERV-LTR sequences of each locus were aligned using ClustalW^38^, and a phylogenetic tree was constructed using the maximum likelihood method in MEGA X^39,40^. Phylogenetic trees and alignments were visualized using FigtTee v1.4.4 (http://tree.bio.ed.ac.uk/software/figtree/) and Mview v1.67^41^, respectively.

## Supplementary Information

Supplementary Information 1.

Supplementary Information 2.

Supplementary Information 3.

Supplementary Information 4.

Supplementary Information 5.

## Data Availability

The LTR sequences of each locus and each junglefowl are listed in Table S4. The datasets used and/or analysed during the current study available from the corresponding author on reasonable request.
